# Virological and Immunological Outcomes in Rhesus Monkeys after Exposure to Dengue Virus–Infected *Aedes aegypti* Mosquitoes

**DOI:** 10.4269/ajtmh.19-0633

**Published:** 2020-05-18

**Authors:** Lauren B. Carrington, Alongkot Ponlawat, Chanyapat Nitatsukprasert, Patcharee Khongtak, Piyanate Sunyakumthorn, Christine A. Ege, Rawiwan Im-erbsin, Kesara Chumpolkulwong, Butsaya Thaisomboonsuk, Chonticha Klungthong, In-Kyu Yoon, Damon Ellison, Louis Macareo, Cameron P. Simmons

**Affiliations:** 1 Oxford University Clinical Research Unit (OUCRU), Wellcome Trust Asia-Africa Programme, Ho Chi Minh City, Vietnam;; 2 Nuffield Department of Medicine, University of Oxford, Oxford, United Kingdom;; 3 Armed Forces Research Institute of Medical Sciences (AFRIMS), Bangkok, Thailand;; 4 Institute for Vector-Borne Diseases, Monash University, Melbourne, Australia

## Abstract

This study describes the natural history of dengue virus (DENV) infection in rhesus monkeys exposed to the bites of DENV-infected *Aedes aegypti* mosquitoes. Dengue virus–infected mosquitoes were generated by either intrathoracic inoculation or by oral feeding on viremic blood meals. Each of the six rhesus monkeys that were fed upon by intrathoracically infected mosquitoes developed non-structural protein 1 (NS1) antigenemia and an IgM response; viremia was detected in 4/6 individuals. No virological or immunological evidence of DENV infection was detected in the three monkeys exposed to mosquitoes that had been orally infected with DENV. These results demonstrate the utility of mosquito-borne challenge of rhesus monkeys with DENV.

## INTRODUCTION

Dengue is the most widespread arthropod-borne viral disease in the world. The causative agent, dengue virus (DENV), is transmitted in a human–mosquito–human transmission cycle, primarily involving *Aedes aegypti* mosquitoes. Intervention strategies aimed at inhibiting the transmission cycle of DENV between humans and mosquitoes are at various stages of testing. One of these strategies involves the use of *Wolbachia* to reduce the vector competence of *Ae. aegypti* populations for medically important arboviruses.^1–3^ Conventionally, researchers assess mosquito vector competence in an in vitro transmission (IVT) assay. The IVT assay involves the restraint and subsequent collection of saliva from individual mosquitoes^4^ which is then tested for the presence of virus, using either direct or indirect detection methods.^5–10^

It is unknown how well the laboratory-based IVT assay correlates with the actual transmission potential, that is, the probability of virus transmission during the bite of an infected mosquito on a susceptible host. Nonhuman primates (NHPs) offer a model animal system that can be used to validate and calibrate IVT assays. Rhesus macaques (*Macaca mulatta*) are susceptible to DENV infection and develop a viremia and immune response but do not develop signs or symptoms of infection.^11–16^ Recent work on Zika virus (ZIKV) infection in macaques demonstrates that the mode of virus delivery can alter the course of infection. Relative to infection via subcutaneous inoculation, delivery of virus through the bite of a ZIKV-infected *Ae. aegypti* prolonged the peak viremia in the animals and increased the virus sequence heterogeneity in the resulting virus population.^17^ Comparable studies of viral kinetics in response to DENV delivered by mosquito bites have not been performed to date. In the only identified study in which NHPs were infected via the bite of infectious mosquitoes,^16^ the infection outcome was measured serologically 28 days after exposure; viremia and NS1 antigenemia profiles were not measured.

This study was motivated by the desire to establish a reliable mosquito-to-NHP infection model that would subsequently allow us to measure the efficacy of *Wolbachia* strains to block transmission. In doing so, we aimed at calibrating the laboratory-based IVT assay to result in the NHP infection model.

## METHODS

### Study outline.

Three independent experiments were conducted. Each experiment involved three rhesus macaques, each directly fed upon by three DENV-infected *Ae. aegypti*. Some variations in the methodological approach between the first and subsequent experiments were necessary, and these are described in the following appropriate sections (Mosquito rearing and origin, and Viral infection of mosquitoes [day −14]).

**Figure 1.**
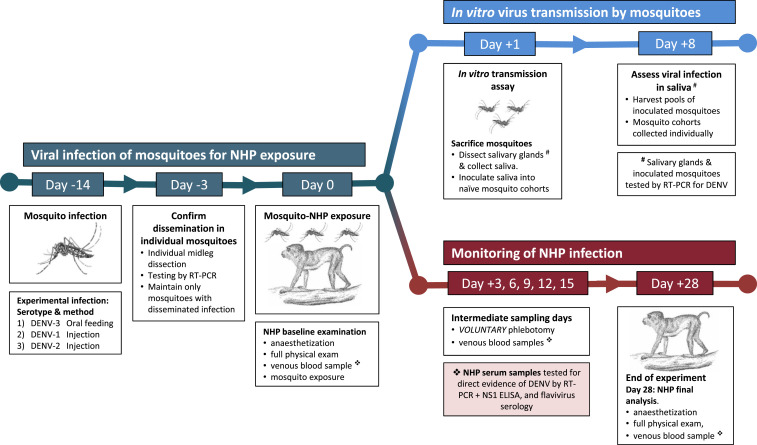
Overview of study design illustrating the infection of mosquitoes, followed by the independent assessments of in vitro transmission in mosquitoes and NHP infection over a 28-day time course. The schematic diagram illustrates the infection and sampling of mosquitoes and NHPs, sampling time points, and steps involved in each part of the study. Viral infection and incubation of mosquitoes took place from day 14–day 0 (in green, left hand side). The top branch of the diagram (in blue) shows the major steps involved in the IVT assay. The IVT assay was performed in parallel with the NHP infections, shown on the bottom branch of the figure (in red). Voluntary blood samples were collected from NHPs to test for evidence of dengue virus infection. Each experiment involved three NHPs, each fed upon by three mosquitoes, and the whole experiment was replicated three times (meaning a total of nine NHPs and 27 mosquitoes). IVT = in vitro transmission; NHP = nonhuman primate.

A schematic diagram of the study design is depicted in Figure 1. In brief, mosquitoes were first infected with virus on day −14, either by oral challenge using viremic blood from an acute dengue patient (as in Experiment 1) or by direct inoculation of virus into the mosquito (Experiments 2 and 3). Two weeks later, DENV-infected mosquitoes were allowed to feed on NHPs, representing day 0. For the next 15 days, NHPs were monitored with intermittent blood sampling at 3-day intervals, coupled with a final day 28 time point to resolve serological status. Every blood sample collected was tested by DENV polymerase chain reaction (PCR), NS1 ELISA, and IgM/IgG ELISA tests. To compare outcomes in the macaque infection model and the IVT assay, the day following the NHP–mosquito exposure (day 1), we tested saliva and salivary glands from each of the nine mosquitoes that fed upon NHPs using our standard IVT protocol.^5,6,10^

### Ethical considerations.

This study was reviewed and approved by the IACUC and Biosafety Review Committee at the Armed Forces Research Institute for Medical Science (AFRIMS), an AAALAC International–accredited facility (protocol number 14-04). All NHP-related research were conducted in accordance with Thai laws; the Animal Welfare Act; all applicable U.S. Department of Agriculture, Office of Laboratory Animal Welfare, U.S. Department of Defense guidelines; and EU standards for NHP research, including housing for NHPs in European ETS123 standard cages. Nonhuman primates were phlebotomized voluntarily during the course of the study (days 3, 6, 9, 12, and 15); only the first and final blood samples (days 0 and 28) were performed under anesthesia, in addition to a full physical examination. All veterinary care was administered by qualified AFRIMS veterinarians. The single human viremic blood sample used in this study was obtained via an ongoing study conducted by the AFRIMS (approval number WRAIR#2093) granted by the Walter Reed Army Institute of Research Institutional Review Board.

### Experimental preparation.

#### Nonhuman primate preselection for experimentation.

Approximately 6–12 months before experimentation, a number of individuals from the *M. mulatta* colony maintained at the AFRIMS were screened for potential inclusion in the experiment. Individuals of both sexes were prescreened to ensure they weighed more than 3.2 kg and were confirmed *Flavivirus* naive, using a hemagglutination inhibition assay (HIA). Naive individuals were recruited for subsequent training for voluntary phlebotomy (in accordance with the European Union regulations for working with NHPs), a process taking 4–6 months. A total of nine NHPs, all of which could be routinely phlebotomized safely, were finally enrolled in the study.

#### Mosquito rearing and origin.

The mosquitoes in the first experiment were field-derived (*F*_0_), originating from the field in Nha Trang, central Vietnam. These mosquitoes were collected as eggs from ovitraps, as described in Carrington et al.^10^ Eggs were then delivered to the AFRIMS, Bangkok, for rearing and experimental use. In the latter two experiments, we used a field strain of Thai *Ae. aegypti*. These mosquitoes originated from Kamphaeng Phet Province, collected from ovitraps in 2016, and colonized for one generation, before the *F*_1_ generation was used in the experiments.

All mosquitoes used in the experiments were reared under standard laboratory conditions, at 26 ± 1°C, under a 12:12-hour light: dark cycle. Larvae were reared at a density of approximately 300 larvae per 29 × 39 × 4.5-cm plastic tray, filled with 2.5 L of dechlorinated water. They were fed daily with 0.8 g of fish food pellets (C.P. Hi Pro^®^, Perfect Company Group Co. Ltd., Bangkok, Thailand).

Adult colonies (where appropriate) were maintained at the AFRIMS with human blood. Human blood purchased from the Thai Red Cross was used to feed *Ae. aegypti* in our mosquito-rearing insectary. The *Ae. aegypti* colony was allowed to feed uninterrupted on the human blood for 1 hour using the artificial membrane technique and was maintained at 25 ± 2°C and 80% relative humidity.

#### Viral infection of mosquitoes (day −14)

On emergence as adults, a surplus of mosquitoes were maintained on 10% sucrose solution ad libitum until which time they were infected with DENV (either by oral feeding or inoculation).

In Experiment 1, mosquitoes were orally challenged with a patient-derived blood meal via artificial membrane feeders. The patient, suffering from a DENV-3 infection, was in their second day of illness at the time of enrollment. Plasma RNAemia was estimated at 3 × 10^8^ genome copies/mL, according to quantitative Reverse Transcription (qRT)-PCR. Because of difficulties with patient recruitment after the first experiment, we were forced to infect mosquitoes parenterally in the remaining two experiments, using cell culture–grown virus. Dengue virus-1 and DENV-2 were used in Experiments 2 and 3, respectively. Mosquitoes were inoculated with ∼1 μL of DENV-1 (00442/05 B isolate, passaged 1 time in *Toxorhynchites splendens* mosquitoes, and five times in C6/36 cells) and DENV-2 (00210/15 isolate, passaged five times in C6/36 cells). The titer of the virus used for inoculation in both experiments was 4 × 10^6^ plaque forming units (PFU)/mL (measured in rhesus monkey kidney cells).

After mosquitoes were exposed to virus, they were housed in paper cups (9-cm height × 8-cm diameter) at a density of 15 females per cup and were maintained with 10% sucrose at 25 ± 1°C for 14 days.

#### Testing for disseminated infection in virus-exposed mosquitoes (day −3)

Virus-exposed mosquitoes were analyzed 3 days before being allowed to feed on NHPs, to determine whether the mosquitoes had a disseminated viral infection. A single midleg was dissected and placed in 150 µL of RPMI medium supplemented with 10% fetal bovine serum. Leg samples were ground and homogenized using a bullet blender (Next Advance, New York, NY) at a speed of 8 for 3 minutes. Homogenized samples were tested by DENV qRT-PCR. After midleg dissection, mosquitoes were maintained individually in the new containers, and their path through the experiments could be traced with their unique identity. Only mosquitoes that tested PCR positive for DENV in leg samples were retained for possible feeding on the NHPs. Those mosquitoes with the highest titer leg infections were selected for the NHP–mosquito exposures, to be conducted on the study day 0. An excess of mosquitoes were prepared to ensure any un-engorged female could be replaced with a fresh mosquito as needed.

#### Infection of NHPs with direct mosquito bite (day 0)

To enable mosquito feeding in a controlled manner, NHPs were anesthetized with ketamine by a registered veterinary doctor. Eight milliliters of venous blood was collected before mosquito exposure to reestablish a negative baseline *Flavivirus* serology.

For each experiment, three anesthetized NHPs were each exposed to three mosquitoes with a PCR-confirmed disseminated DENV infection. The mosquitoes chosen to use in the exposures were those with the highest viral concentration in the leg tissues. Mosquitoes were allowed 15 minutes to feed to repletion on the skin of the NHP; if they did not feed at that time, they were replaced with the mosquitoes with the next highest leg titer. We closely monitored and observed mosquito’s probing and feeding activity at all times; when mosquitoes were inactive and did not probe/feed on an NHP, they were replaced. A maximum of three mosquitoes fed to repletion on each NHP, and after feeding, mosquitoes were retained for subsequent in vitro testing (see next section).

#### In vitro virus transmission by mosquitoes (days 1 and 8)

We performed the standard IVT assay using the same mosquitoes that had fed upon NHPs the day prior. In performing the IVT assay 1 day later (day 1), we aimed at ensuring mosquitoes had replenished their saliva.

Mosquitoes were killed in the conventional laboratory-based IVT assay, with saliva collected from de-legged/de-winged mosquitoes, before subsequent salivary gland dissection. Salivary glands were tested directly by RT-PCR to determine the presence of viral RNA. Each saliva sample was inoculated into 5–6 virus-naive *Ae. aegypti* mosquitoes, which were then incubated for 7 days (day 8), allowing for any virus present in the saliva to amplify within the inoculated mosquitoes. At the completion of the incubation period, the inoculated *Ae. aegypti* from a single index mosquito were pooled and tested for the presence of virus by PCR (see Diagnostics section). A positive result was interpreted, as a mosquito that had transmission potential in the IVT assay. The unique identity and infection status of each individual were then associated with the infection status of the NHP on which it fed.

#### Routine monitoring of NHPs for infection (day 3 to 28)

After the mosquito–NHP exposure on day 0, NHPs were monitored with voluntary blood draws of 1–2 mL volume, every 3 days until day 15. Blood was collected from the cephalic vein in the arm. As all blood draws were voluntary, if an NHP was refused to provide a blood sample on a given day, a second attempt would be made on that day; if the second attempt to draw blood was refused, that blood sample would be forsaken. The final blood draw, taken on day 28, was performed while the NHP was under anesthesia, along with a routine physical examination to ensure the NHP remained in good health. As with the first blood sample, this final 8 mL was collected from the saphenous vein in the leg. Serum was separated from each blood sample and prepared into three aliquots, assigned for each screening assay. We tested for 1) DENV NS1 antigen, 2) IgM and IgG antibody responses to both DENV and JEV antigens, and 3) DENV RNA in a nested RT-PCR.

### Diagnostic testing of samples.

#### Mosquito sample processing

At respective time points, mosquito tissues (legs, salivary glands, or pooled inoculated mosquito samples) were homogenized in RPMI 1640 (Sigma Aldrich, St. Louis, MO) medium mixed with 10% FBS. A 140 µL sample of the homogenate was used for nucleic acid extraction using the QIAamp Viral RNA Mini Kit (Qiagen, Hilden, Germany). All mosquito tissues were screened by RT-PCR for DENV infection as described in the Molecular Diagnostics section. Measurements for mosquito samples were returned in genome equivalents (GE)/150 µL of the homogenate for mosquitoes.

#### Nonhuman primates sample processing

A serum sample was collected from each NHP for HIA testing between 10 and 52 days before experimental exposure to mosquitoes. Samples were tested by HIA using the method described in Clarke and Casels.^18^ Samples with a titer < 10 were considered negative for past *Flavivirus* infection.

Postexposure to mosquitoes, sera from the NHPs were tested for NS1 antigen in the blood using BioRad’s NS1 ELISA kit, following the manufacturer’s instructions. Results are presented in the sample-to-cutoff ratio, where values greater than 1 were considered positive and those less than 0.5 were considered negative. Values between 0.5 and 1 were considered equivocal; the first time an equivocal result was observed, the test was performed a second time. The second result was used as the final result. Antibody responses were investigated using an enzyme immune assay (EIA), as per Kato et al.^19^ The IgM and IgG results were considered positive if there was a 4-fold rise in EIA units between days 0 and either day 14 or 28. The same cutoff was used for both DENV and JEV assays. Sera samples were also tested for the presence of viral RNA after RNA extractions using the QIAamp Viral RNA Mini Kit, as per the manufacturer’s instruction. Viral RNA was detected using the TaqMan real-time RT-PCR method as described by Klungthong et al.^20^ Samples were considered positive if the cycle threshold values were less than 40. Viral titers were calculated for each sample and reported in GEs/mL of serum.

### Data analysis.

As the aim of this work was to explore the reliability and features of DENV infection delivered by direct mosquito bites to NHPs, we provide a descriptive summary of the kinetics of RNAemia, NS1 antigenemia, and IgM/IgG antibodies. We also describe the prevalence of virus transmission using the IVT assay relative to the NHP infection model. All graphical outputs were produced using R software (RStudio Team 2015, Boston, MA).

## RESULTS

We performed three DENV infection experiments, each with three NHPs fed upon by three mosquitoes (a total of nine rhesus monkeys and 27 mosquitoes). On each of the scheduled sampling days, all NHPs volunteered blood samples, meaning the sampling regime (Figure 1) was complete for analysis. In the first experiment, NHPs fed upon by mosquitoes that had been orally infected with DENV-3 did not result in infection (negative for NS1, RT-PCR for viral RNA, and IgM/IgG ELISA).

The subsequent two experiments, each using mosquitoes that had been intrathoracically inoculated with DENV-1 and DENV-2, both resulted in a 100% attack rate. Descriptive results of each experiment for both the mosquito and the associated NHPs, are detailed in the following texts, alongside a summary of the results for all three experiments presented in Table 1.

**Table 1:** Summary of test results for mosquitoes and NHPs after infection with DENV-1, 2, or 3

Summary of experimental details	Mosquito results (from RT-PCR)					NHP results (qualitative summary across all time points)				
	Mosquito replicate	Index mosquito number*	Dissected legs	Dissected salivary glands	Pool of inoculated mosquitoes	NHP ID	Viremia (RT-PCR)	NS1 antigenemia (enzyme immune assay)	DENV IgM (ELISA)	DENV IgG (ELISA)
Experiment 1: Patient-derived blood meal administered by oral challenge for *F*_0_ mosquitoes from Vietnam	1	14	Positive	Positive	Positive					
	2	20	Positive	Positive	Negative	R762	Negative	Negative	Negative	Negative
	3	27	Positive	Positive	Positive					
	4	Gr3.1	Positive	Positive	Negative					
	5	Gr3.2	Positive	Positive	N/A^¶^	R1014	Negative	Negative	Negative	Negative
	6	21	Positive	Positive	N/A^¶^					
	7	Gr2.1	Positive	Positive	Positive					
		8	10	Positive	Positive	Negative	R1054	Negative	Negative	Negative	Negative
		9	19	Positive	Positive	Positive					
Experiment 2: DENV-1 cell culture–grown virus inoculated into the thorax of *F_1_* mosquitoes from Thailand	10	6	Positive	Positive	Positive					
	11	7	Positive	Positive	Positive	R1112	Positive	Positive	Positive	Positive
	12	20	Positive	Positive	Positive					
	13	1	Positive	Positive	Positive					
	14	14	Positive	Positive	Positive	R1138	Positive	Positive	Positive	Positive
	15	16	Positive	Positive	Positive					
	16	45	Positive	Positive	Positive					
		17	40	Positive	Positive	Negative	R1141	Positive	Positive	Positive	Positive
		18	39	Positive	Positive	Positive					
Experiment 3: DENV-2 cell culture–grown virus inoculated into the thorax of *F_1_* mosquitoes from Thailand	19	26	Positive	Positive	Positive					
	20	42	Positive	Positive	Positive	R710	Positive	Positive	Positive	Positive
	21	48	Positive	Positive	Positive					
	22	22	Positive	Positive	Positive					
	23	35	Positive	Positive	Positive	R1007	Negative	Positive	Positive	Positive
	24	36	Positive	Positive	Positive					
	25	44	Positive	Positive	Positive					
		26	58	Positive	Positive	Positive	R1146	Negative	Positive	Positive	Positive
		27	61	Positive	Positive	Positive					

DENV = dengue virus; NHP = nonhuman primate; ¶ = no mosquitoes from these cohorts survived to the day of harvesting. Mosquito tissue results represent the qualitative test result. NHP results represent the qualitative result from respective tests, with at least one “positive” test result across any time point for a particular test being considered as a positive result for that assay overall.

* Mosquitoes listed with “GrX.X” were initially grouped together in a single cup for the actual mosquito–NHP exposure. Therefore, their individual leg titers could not be linked to their eventual transmission potential because their unique identity was unlinked when they were grouped. All other mosquitoes can be traced from the beginning to the end.

### Experiment 1: Mosquito infection via oral challenge with patient-derived DENV-3 blood meal.

**Figure 2.**
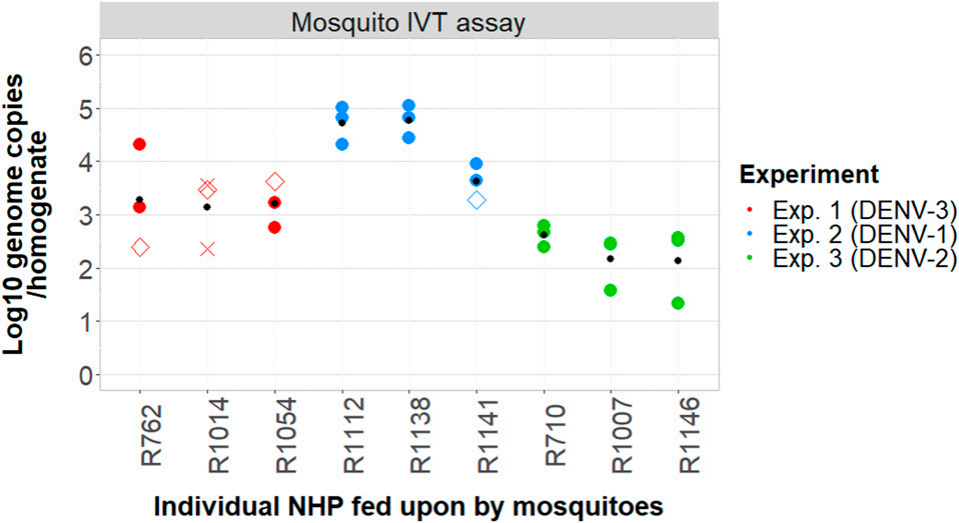
Infection results for mosquitoes tested in the in vitro transmission (IVT) assay, indicating the viral load in the salivary glands and the transmission status of each mosquito. Each data point represents the log_10_ viral load of the salivary glands from a single mosquito, as measured by RT-PCR. These points are plotted against the nonhuman primate (NHP) on which they fed (3 mosquitoes for each NHP). Mosquitoes were tested for IVT 1 day after they had fed upon the NHPs. Each data point represents a single mosquito that was exposed to virus and fed upon one of the NHPs. Closed colored circles (●) represent mosquitoes with evidence of virus in their saliva in the IVT assay, open colored diamonds (◇) represent mosquitoes with no evidence of virus transmission, crosses (✕) represent mosquitoes for which no data are available about their transmission status (i.e., all the mosquitoes that were inoculated with their saliva died before sampling). The smaller black dot (•) represents the average viral titer in the salivary glands of those three mosquitoes that fed upon each respective NHP.

**Figure 3.**
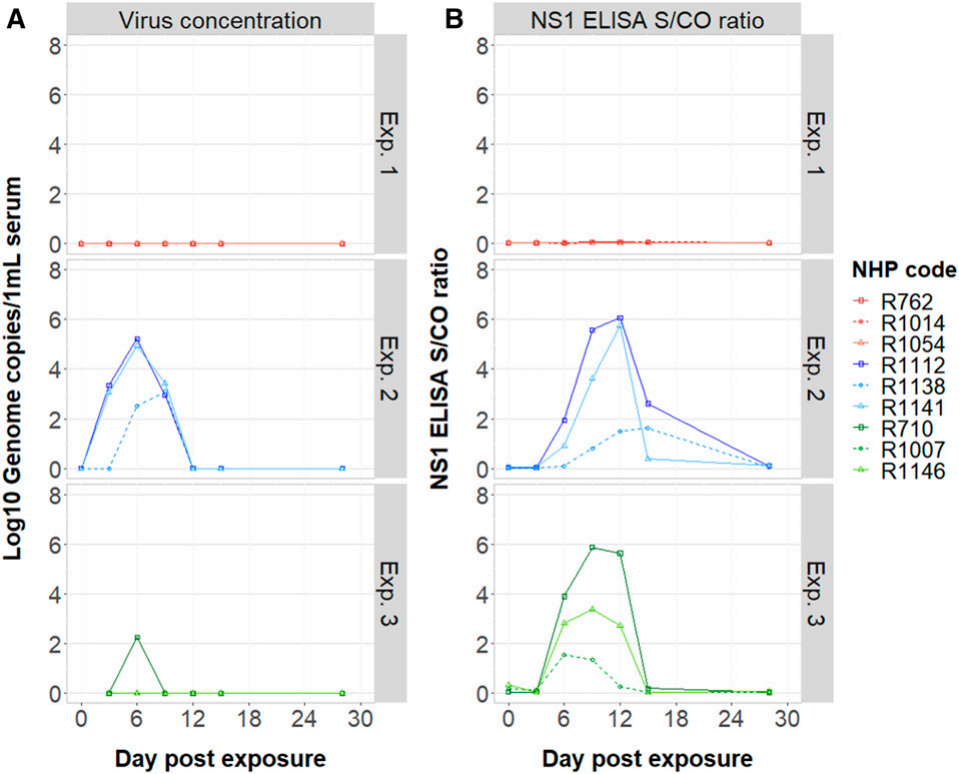
Temporal virological responses observed in nonhuman primates (NHPs) exposed to dengue virus (DENV) via direct mosquito bites. Data are stratified by experiment. (**A**) Changes in viral concentration in the serum, as measured by RT-PCR. (**B**) Detection of DENV NS1 antigenemia (measured by enzyme immune assay). The signal-to-cutoff (S/CO) ratio of the assay is plotted as a function of the day at which the NHPs were sampled.

**Figure 4.**
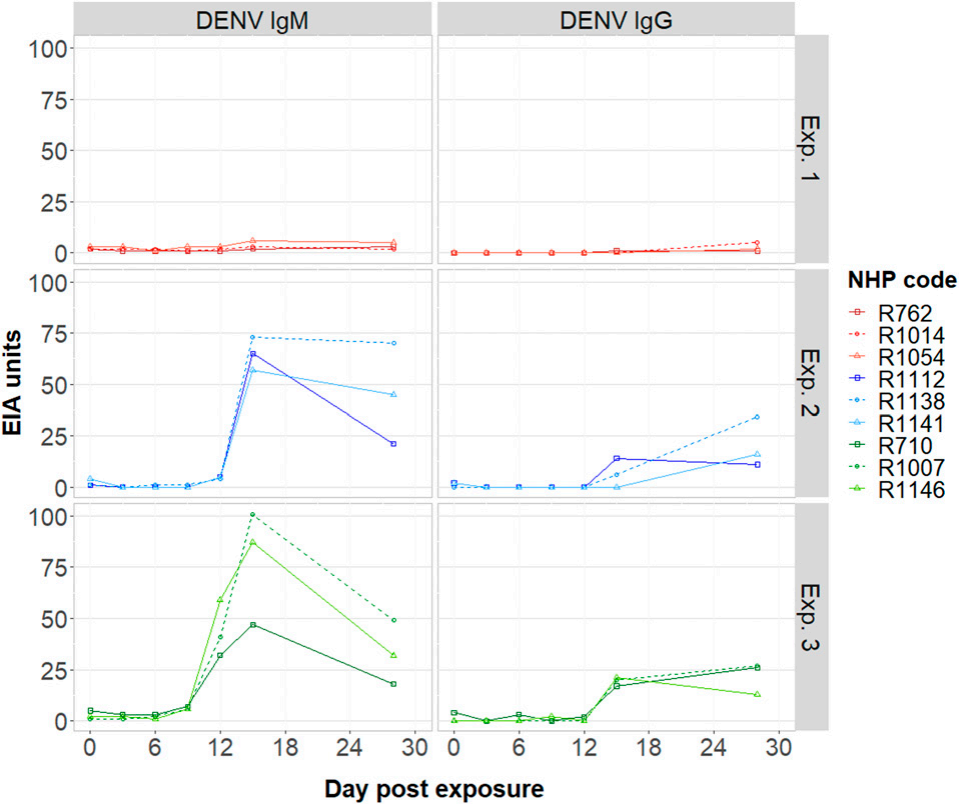
Temporal immunological responses of nonhuman primates (NHPs) after infection with dengue virus (DENV), stratified by experiment. The figure shows the serological changes, measured by ELISA, for DENV IgM/IgG according to the day of NHP sampling.

Mosquitoes in Experiment 1 were orally infected with a patient-derived DENV-3 viremic blood meal (> 10^8^ viral genome copies/mL). A total of 96.5% (28/29) of mosquitoes developed a disseminated leg infection in pre-exposure screening. The final cohort of nine mosquitoes that fed upon the NHPs had DENV-positive salivary glands, with a minimum viral load of 4.9 × 10^3^ GE copies of virus/sample (Figure 2). Most of these nine mosquitoes also had saliva that tested positive for DENV (Table 1). However, we failed to detect evidence of DENV infection in any of the NHPs (Table 1, Figures 3 and 4).

### Experiment 2: Mosquito infection via intrathoracic inoculation of cell-cultured DENV-1.

In Experiment 2, mosquitoes were injected with ∼1 µL of 4 × 10^6^ genome copies/mL DENV-1 serotype. All 50 index mosquitoes inoculated with cultured virus developed disseminated leg infections. The lowest viral load among the dissected salivary glands from these mosquitoes was 4 × 10^4^ GE virus/homogenate. Eight of nine mosquitoes also had saliva that tested positive for DENV (Table 1). All three NHPs had virological and serological evidence of DENV infection after being fed upon. Two individuals (R1112 and R1141) had detectable RNAemia on days 3, 6, and 9, with peak titers observed on day 6 (∼10^5^ GE virus/1 mL serum). Detection of RNAemia in NHP R1138 was observed on days 6 and 9 only and peaked ∼2 logs lower than the other two NHPs (Figure 3). Nonhuman primate R1112, which had the highest RNAemia overall, had detectable NS1 circulating on days 6, 9, 12, and 15. Nonhuman primate R1141 was NS1 antigenemic on days 9 and 12 only. Nonhuman primate R1138, which had the delayed onset of RNAemia, was also delayed in the detection of DENV NS1 (Figure 3). In all individuals, DENV-reactive IgM was the highest at the day 15 time point, with > 50 EIA units (but may have peaked either before or after), but remained detectable at day 28. Dengue virus–reactive IgG was detected on either day 15 or 28 (Figure 4).

### Experiment 3: Mosquito infection via intrathoracic inoculation of cell-cultured DENV-2.

In Experiment 3, mosquitoes were again inoculated with virus but this time with DENV-2. Consistent with the previous injection experiment, 100% of inoculated mosquitoes developed disseminated leg and salivary gland infections. Viral load in the mosquito salivary glands ranged between 4.5 × 10^2^ and 1.3 × 10^4^ GE virus/homogenate. Despite the average salivary gland virus titer of these nine engorged mosquitoes being lower than that of the nine engorged mosquitoes in Experiment 1 (Figure 2), all females in this experiment tested positive for virus in their saliva in the IVT assay. All three NHPs developed DENV infection. NS1 was positive on days 6, 9, and 12 in all individuals (Figure 3), and although IgM became detectable on day 12, its peak was on day 15. The highest IgM measurements were observed in the single individual that had a detectable RNAemia (R710; 1.7 × 10^2^ GE virus/1 mL serum; Figure 4). IgG was detected on days 15 and 28 in all three individuals.

## DISCUSSION

One of the aims of this study was to characterize the NHP response to virus infection by direct mosquito bites, after feeding on a human patient–derived blood meal. Although unsuccessful in this particular goal, we were able to infect six NHPs following the direct bites of mosquitoes parenterally infected with virus, and characterize the viral kinetics and immune response profiles in these NHPs. In doing so, we report our descriptive comparison of the IVT and NHP infection.

In total, 100% of NHPs were DENV-infected after receiving bites of *Ae. aegypti* mosquitoes that had been infected by intrathoracic inoculation. RNAemia was detected in all three NHPs exposed to DENV-1 and one NHP exposed to DENV-2. Interestingly, the two individuals without DENV-2 RNAemia had the highest concentrations of anti-DENV antibodies. Although our 3-day intervals between sampling may have led us to miss a small window in which RNAemia may have been detectable, it is plausible that in these animals, a robust antibody response substantially reduced the magnitude and duration of the viremia.^21^ As also observed in human patients infected with DENV-2,^22^ we noted individual NHPs with low NS1 also had lower (or non-detectable) levels of RNAemia.

Overall, the onset and duration of RNAemia among NHPs infected by direct mosquito bites are similar to those subcutaneously inoculated with DENV.^11,23^ Likewise, Hickey et al.^15^ demonstrated that after inoculating rhesus monkeys with 10^5^ PFU/mL of each of four DENV serotypes, IgM titers were at maximal levels (based on the time points tested) on either day 10 or 14. In our own experiments, a quick increase in IgM levels and peak were observed, in response to DENV-1 and DENV-2 infections, around day 15.

Surprisingly, when we used *Ae. aegypti* that had been infected with DENV-3 by oral feeding on the dengue patient’s blood, we failed to detect any evidence of infection in the three NHPs. This was despite all nine index mosquitoes having DENV-infected salivary glands and four of seven mosquitoes possessing infectious virus in their saliva. Pinpointing contributing factor(s) to explain this outcome is challenging, however, given a number of factors were changed between experiments, including the use of clinical versus cell-cultured virus isolates, mosquito infection via oral feeding versus intrathoracic inoculation, different virus serotypes and virus titers, and different origins of the mosquito populations. Plausibly, these three NHPs may have been inherently resistant to DENV infection (although serologically they were *Flavivirus* naive). Subsequent DENV challenge of the NHPs in question by inoculation with cultured virus could help test this hypothesis. DENV-3 is able to infect NHPs^23^; however, the particular DENV-3 in question might have been inherently less infectious than the DENV-1 and DENV-2 we used. If we had used more animals per group or used more infected mosquitoes per animal, we might have observed a different outcome.

A limitation of this pilot study is that it is unknown whether a single mosquito or multiple mosquitoes are responsible for delivering the infectious bite(s) to the naive NHPs. Given our inability to reliably infect NHPs using DENV-infected mosquitoes, further work is still needed to develop an epidemiologically relevant human–mosquito–NHP infection model capable of testing the efficacy of novel mosquito interventions such as *Wolbachia*. Despite this, our work identifies reliable time points for screening DENV infection in this species after being fed upon by DENV-1- and DENV-2-infected mosquitoes to maximize the detection of viremia, NS1 antigenemia, and DENV-reactive antibodies.
